# Association between the Epidermal Growth Factor +61G/A Polymorphism and Glioma Risk: A Meta-Analysis

**DOI:** 10.1371/journal.pone.0095139

**Published:** 2014-04-16

**Authors:** Xin Chen, Guang Yang, Daming Zhang, Weiguang Zhang, Huichao Zou, Hongbo Zhao, Xinjian Zhang, Shiguang Zhao

**Affiliations:** 1 Department of Neurosurgery, The First Affiliated Hospital of Harbin Medical University, Harbin, Heilongjiang Province, People's Republic of China; 2 Institute of Brain Science, Harbin Medical University, Harbin, Heilongjiang Province, People's Republic of China; The Ohio State University Medical Center, United States of America

## Abstract

**Background:**

Gliomas account for almost 80% of primary malignant brain tumors. Epidermal growth factor (EGF) is an interesting research candidate in which to look for genetic polymorphisms because of its role in mitogenesis and proliferation. Extensive studies have found that a single nucleotide polymorphism (SNP) +61G/A (rs4444903) in the EGF gene is associated with the susceptibility of glioma, however, the results have been controversial. Furthermore, the association between EGF +61G/A polymorphism with the development and grade progress of glioma has not been established.

**Methods:**

We examined the association of EGF +61G/A polymorphism and glioma by performing a meta-analysis. Nine studies testing the associations between EGF +61G/A polymorphism and risk of glioma with 1758 cases and 2823 controls were retrieved. Odds ratios (ORs) and 95% confidence intervals (CIs) were used to assess the strength of the association. The pooled ORs were performed for the allele model, codominant model, dominant model, and recessive model, respectively.

**Results:**

Overall, this meta-analysis showed significant associations between the EGF +61G/A polymorphism and glioma susceptibility in all four genetic models. However, in the stratified analysis by the grade of glioma, we only found this association existed in patients with Grade IV glioblastoma, but not in patients with Grade I-III glioma. We further compared EGF +61G/A polymorphism in patients with glioblastoma and Grade I-III glioma accordingly, the stronger association between the EGF +61G/A polymorphism and the malignancy of glioma was found.

**Conclusions:**

The results of this meta-analysis suggested that the EGF +61G/A polymorphism is associated with both the susceptibility of glioma and the malignance of glioma.

## Introduction

Gliomas are the most common primary tumors in the central nervous system [1]. Gliomas have complex cellular composition and inherent resistance to the treatments. Carcinogenesis of glioma is a complex pathological process, in which environmental and genetic factors interact with each other [2]. Ionizing radiation is a known factor which is clearly associated with brain tumors development risk [3]. However, many patients without known environmental factors eventually developed glioma. Therefore, genetic predisposition may contribute to the process of glioma carcinogenesis. According to the World Health Organization (WHO) criteria, gliomas are histologically classified into Grades I to IV [4]. The higher the glioma grade is, the more resistant to the treatments and the shorter median survival time the patient will have [5]. The genetic heterogeneity of glioma also accounts for the current limitations in predicting patient prognosis based on histologic classification of glioma grade alone [6], [7] and suggests that classification of gliomas from the same grade according to their genetic phenotype will provide a more accurate prediction of both patient survival time and the response to the therapy [8], [9].

The epidermal growth factor (EGF) gene, which is a member of the EGF superfamily, has been demonstrated to activate cell proliferation and stimulate mitogenesis in epidermal tissues [10]. It is a natural focus of interest in cancer studies because of its ability to influence mitogenesis and proliferation, and its overexpression is commonly seen in human cancers, such as glioma, pancreatic cancer, breast cancer and hepatocarcinoma [11], [12]. Some studies reported that EGF plays a critical role in gliomagenesis by influencing cell proliferation, inhibiting apoptosis, and inducing cell differentiation, via binding to its specific receptor, the EGF receptor (EGFR) [13], [14]. Previously, Shahbazi et al. identified a functional single nucleotide polymorphism involving guanine (G) to adenine (A) substitution at position 61 in the 5′-untranslated region of the EGF gene (rs4444903), and the substitution increased production of EGF in cultured peripheral blood mononuclear cells [15]. Thus, this genetic polymorphism may contribute to interindividual differences of EGF expression and subsequently the tumor predisposition and aggressiveness.

To date, several studies have found the association between the EGF +61G/A polymorphism with the susceptibility of glioma, however, these studies showed inconsistent results [16]–[19]. Some molecular epidemiological studies suggested that the inconsistency might be caused by the diverse ethnicity of patients [16], [17], however, we hypothesize that the diverse distribution of EGF gene frequencies among the different ethnic groups may cause the inconsistency [20], [21]. In addition, previous researches did not analyze the association in different glioma grade. The comprehensive analysis of glioma grade and genetic phenotype may more accurately predict the prognosis of patients. Therefore, a meta-analysis of nine case-control studies involving 1,758 cases and 2,823 controls was performed to drive a more precise estimation of the association of EGF +61G/A polymorphism with susceptibility to and severity of the glioma.

## Materials and Methods

The procedures of this meta-analysis adhered to the PRISMA Statement guidelines [22] (Supporting Information Checklist S1).

### Literature search strategy for identification of the studies

We did a literature search in online database such as PubMed, Embase and the Chinese knowledge library using the search terms “epidermal growth factor”, “polymorphism”, and “glioma”, along with additional terms such as “EGF, SNP, Glioblastoma, astrocytoma, oligodendroglioma”, and all possible combinations (from January 1, 2000 to September 5, 2013). The search was limited to English-language articles. In order to search more deeply, we reviewed the references of the selected articles to retrieve data that we could have ignored in the initial search. Of the studies with overlapping data published by the same researchers, we selected the most recent ones with the largest number of subjects.

### Inclusion and exclusion criteria

The studies for the meta-analysis were selected when they satisfied the following criteria: studies about EGF +61G/A polymorphism and glioma risk, case-control studies in humans, studies containing useful genotype frequencies or odds ratio (OR) data, studies containing classification of glioma grade. Major exclusion criteria were (a) no control population, (b) no available genotype frequency, (c) no explicit glioma grade classification, and (d) duplication of the previous publication.

### Data extraction

Information was extracted from all eligible articles independently by two of the authors following the criteria listed above (Xin Chen and Guang Yang). Disagreement was discussed and resolved between the two authors.

For each study, the following data were considered: the first author's last name, year of publication, country of origin, ethnicity and numbers of genotyped cases and controls. We did not define any minimum number of patients to include a study in our meta-analysis. In the event that a study presented subpopulations, these were taken to be different studies.

### Statistical analysis

The estimate of association between the EGF +61G/A polymorphism and glioma risk was assessed using the fixed-effect method [23] which calculates the crude ORs and the corresponding 95% CIs for individual studies and the global association. In this meta-analysis, small letter “a” was defined as the minor allele or the mutant allele of EGF +61G/A polymorphism, while capital letter “A” was defined as the major allele or the common allele of EGF +61G/A polymorphism. The pooled ORs were performed for the allele model (a versus A), the codominant model (aa versus AA; Aa versus AA), the dominant model (aa/Aa versus AA), and the recessive model (aa versus Aa/AA), respectively. Stratified analyses were performed by tumor grade. When the test was heterogeneous, the random-effect method [24] was applied.

The analysis of heterogeneity between studies was performed by the Q statistic, with p-values<0.05 indicating significant heterogeneity [25]. Sensitivity was performed by omitting individual studies in order to assess the stability of results. Begg's funnel plots were used to detect publication bias. Additionally, Egger's linear regression test which measures funnel plot asymmetry was also used to evaluate the publication bias, If P<0.05, statistically significant publication bias existed [26]. All analyses were calculated using the STATA Version 11.0 software (version 11; Stata Corporation, College Station, Texas).

## Results

### Characteristics of included Studies

**Figure 1:**
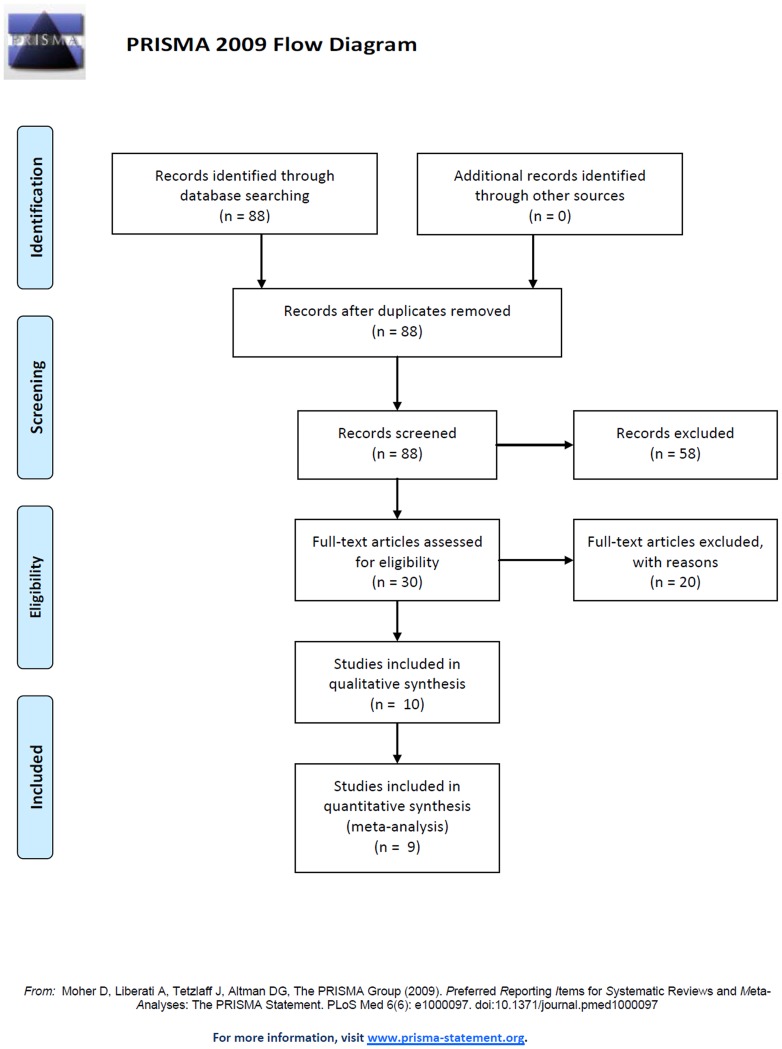
Flow chart of the study selection process.

Figure 1 outlines our study selection process. Briefly, a total of 88 potentially relevant articles were systematically identified after an initial search. After reading the titles and abstracts, we removed 58 articles not examining the association between EGF polymorphism and glioma risk, 11 articles not examining EGF +61G/A polymorphism, and 9 articles belong to review and meta-analysis. After reading full texts of the remaining 10 articles regarding the association between the rs4444903 polymorphism on the EGF gene and glioma, one of these was excluded for not providing allele frequencies in glioma grade subgroup needed for OR calculation [27]. Finally, a total of 9 case–control studies from 5 countries involving 1,758 cases and 2,823 controls were accepted for the pooled analysis. Table 1 presents the main characteristics of these studies.

**Table 1:** Characteristics of individual studies included in meta-analysis.

First author (Year)	Ethnicity (Country)	Genotyping method	Source of control	Case	Control	Case												Control			Controls in HWE
						AA				Aa				aa				AA	Aa	aa	
						I	II	III	IV	I	II	III	IV	I	II	III	IV				
Bao (2010)	Chinese (China)	PCR-RFLP	Hospital	160	320	4	26	17	17	3	25	24	19	2	3	5	15	141	151	28	0.162
Wang (2010)	Chinese (China)	PCR-LDR	Population	672	693	25	103	62	108	28	109	49	119	7	22	5	35	366	276	51	0.917
Liu (2009)	Chinese (China)	PCR-RFLP	Hospital	168	194	7	25	27	16	4	18	35	21	0	6	4	5	92	86	16	0.510
Li (2012)	Chinese (China)	PCR-RFLP	Hospital	180	360	6	23	15	31	7	26	17	33	3	8	4	7	163	176	21	0.003
Pinto (2009)	Caucasian (Brazil)	PCR-RFLP	Hospital	165	200	7	16	3	11	9	20	6	39	6	16	6	26	52	92	56	0.260
Costa (2007)	Caucasian (Portugal)	PCR-RFLP	Hospital	79	570	Na	Na	Na	19	Na	Na	Na	35	Na	Na	Na	25	173	266	131	0.142
Silveira (2012)	Caucasian (Brazil)	PCR-RFLP	Hospital	83	196	Na	Na	Na	24	Na	Na	Na	43	Na	Na	Na	16	40	109	47	0.112
Bhowmick (2004)	Caucasian (USA)	qRT-PCR	Hospital	42	76	Na	Na	Na	11	Na	Na	Na	16	Na	Na	Na	15	27	37	12	0.909
Vauleon (2007)	Caucasian (France)	Real-time PCR	Population	209	214	Na	Na	Na	63	Na	Na	Na	102	Na	Na	Na	44	63	120	31	0.031

Na, not available.

Among the 9 publications, there were five studies of Caucasian descendants [20], [28]–[31] and four studies of Asian descendants [21], [32]–[34]. Gliomas were confirmed histologically or pathologically in all included studies. A classic polymerase chain reaction-restriction fragment length polymorphism (PCR-RFLP) assay was performed in most studies [21], [29]–[32], [34], only one study used polymerase chain reaction-ligation detection reaction (PCR-LDR) method [33], one study used quantitative reverse transcription-PCR (qRT-PCR) technique [28], and one study used Real-time PCR method [20]. The controls were mainly hospital populations and all studies indicated that the distribution of genotypes in the controls were consistent with Hardy-Weinberg equilibrium (HWE) except for two studies [20], [34].

### Association between EGF +61G/A polymorphism and glioma risk

**Figure 2:**
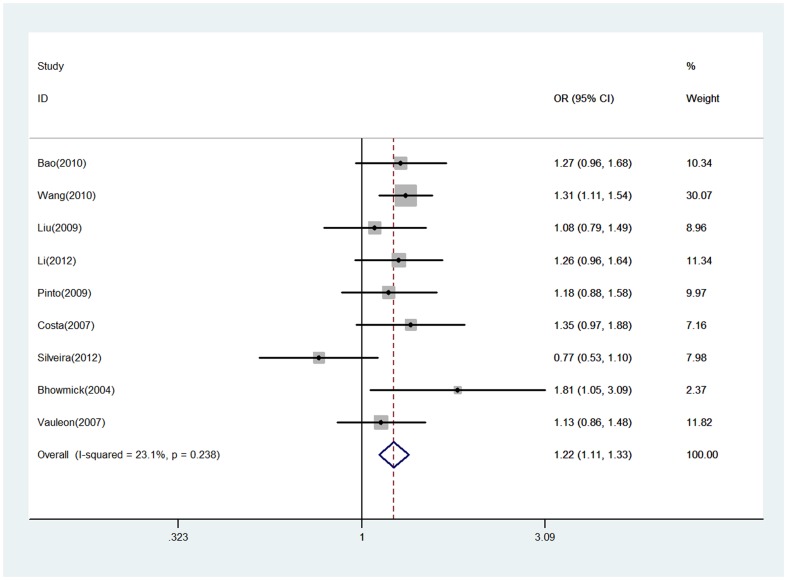
Forest plot of glioma risk for EGF +61G/A polymorphism (a versus A). The squares and horizontal lines correspond to the study-specific OR and 95% CI. The area of the squares reflects the study-specific weight (inverse of the variance). The diamond represents the pooled OR and 95% CI.

**Figure 3:**
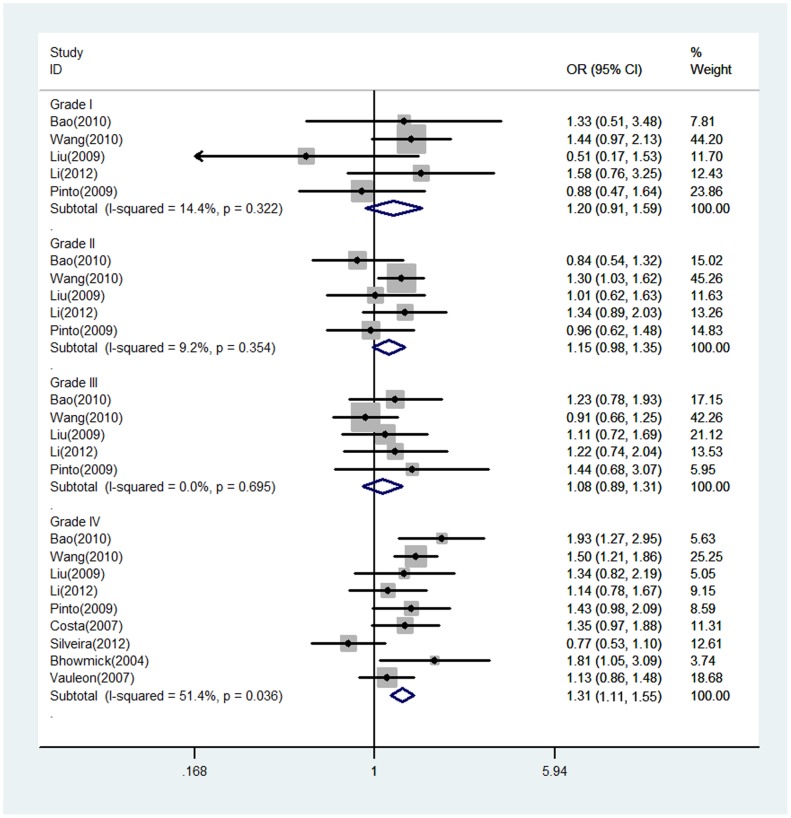
Forest plot of glioma risk for EGF +61G/A polymorphism (a versus A) by glioma grade. The dots and horizontal lines correspond to the study-specific OR and 95% CI. The diamond represents the pooled OR and 95% CI.

The evaluation of association between EGF +61G/A polymorphism and glioma risk are presented in Table 2. There were no significant heterogeneity between studies in all comparisons. Overall, significant associations between the EGF +61G/A polymorphism and glioma risk were observed in all genetic models (a versus A, OR = 1.22, 95% CI = 1.11–1.33; aa/Aa versus AA, OR = 1.21, 95% CI = 1.06–1.38) (Fig. 2 and Fig. S1). However, in the stratified analysis by the grade of glioma, we found the EGF +61G/A polymorphism was significantly associated with increased risk of glioma among patients with glioblastoma (Grade IV) (a versus A, OR = 1.31, 95% CI = 1.11–1.55; aa/Aa versus AA, OR = 1.31, 95% CI = 1.11–1.55), and but not among patients with Grade I-III glioma (Grade I: a versus A, OR = 1.20, 95% CI = 0.91–1.59; aa/Aa versus AA, OR = 1.18, 95% CI = 0.81–1.74; Grade II: a versus A, OR = 1.15, 95% CI = 0.98–1.35; aa/Aa versus AA, OR = 1.16, 95% CI = 0.94–1.43; Grade III: a versus A, OR = 1.08, 95% CI = 0.89–1.31; aa/Aa versus AA, OR = 1.14, 95% CI = 0.88–1.48) (Fig. 3 and Fig. S2).

**Table 2:** Meta-analysis of the association between EGF +61G/A polymorphism and glioma risk.

*Variables*		*n* a	*a vs. A*		*aa vs. AA*		*Aa vs. AA*		*aa/Aa vs. AA (dominant)*		*aa vs. Aa/AA (recessive)*	
			OR(95% CI)	P^b^	OR(95% CI)	P^b^	OR(95% CI)	P^b^	OR(95% CI)	P^b^	OR(95% CI)	P^b^
**case vs. control**	Total	9	1.22[1.11,1.33]	0.238	1.54[1.26,1.88]	0.179	1.12[0.98,1.28]	0.469	1.21[1.06,1.38]	0.387	1.47[1.23,1.75]	0.233
	Grade I	5	1.20[0.91,1.59]	0.322	1.52[0.85,2.73]	0.356	1.10[0.73,1.65]	0.579	1.18[0.81,1.74]	0.44	1.50[0.88,2.55]	0.414
	Grade II	5	1.15[0.98,1.35]	0.354	1.32[0.93,1.90]	0.289	1.11[0.89,1.39]	0.261	1.16[0.94,1.43]	0.28	1.31[0.94,1.82]	0.374
	Grade III	5	1.08[0.89,1.31]	0.695	1.06[0.65,1.73]	0.429	1.16[0.89,1.52]	0.936	1.14[0.88,1.48]	0.882	1.01[0.64,1.60]	0.358
	Grade IV	9	1.31[1.11,1.55]^c^	0.036	1.85[1.29,2.66]^c^	0.033	1.16[0.97,1.38]	0.25	1.31[1.11,1.55]	0.125	1.71[1.27,2.31]^c^	0.048
**Grade IV vs. Grade I-III**		5	1.28[1.08,1.51]	0.207	1.83[1.28,2.61]	0.148	1.19[0.93,1.52]	0.356	1.30[1.03,1.63]	0.343	1.90[1.42,2.54]	0.145

a Number of comparisons; ^b^P value of Q-test for heterogeneity test; ^c^Estimates for random-effects mode.

**Figure 4:**
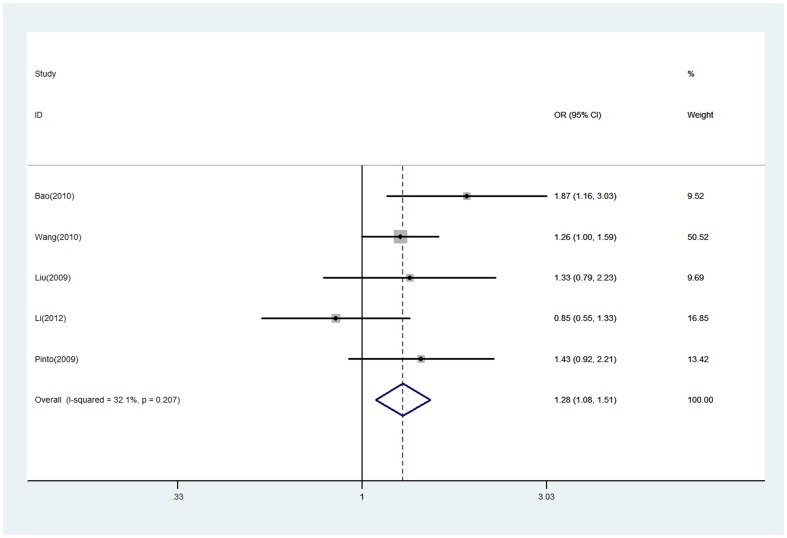
Forest plot of EGF +61G/A polymorphism associated with glioblastoma and Grade I-III glioma risk (a versus A). The squares and horizontal lines correspond to the study-specific OR and 95% CI. The area of the squares reflects the study-specific weight (inverse of the variance). The diamond represents the pooled OR and 95% CI.

At the same time, on the basis of glioma grade subgroup, we further compared the EGF +61G/A polymorphism in patients with glioblastoma and Grade I-III glioma, and found the OR value was greater than 1 (a versus A, OR = 1.28, 95% CI = 1.08–1.51; aa/Aa versus AA, OR = 1.30, 95% CI = 1.03–1.63), in the sense that by contrast to Grade I-III glioma, glioblastoma has the stronger associations with EGF +61G/A polymorphism (Fig. 4 and Fig. S3).

### Sensitivity analysis

**Figure 5:**
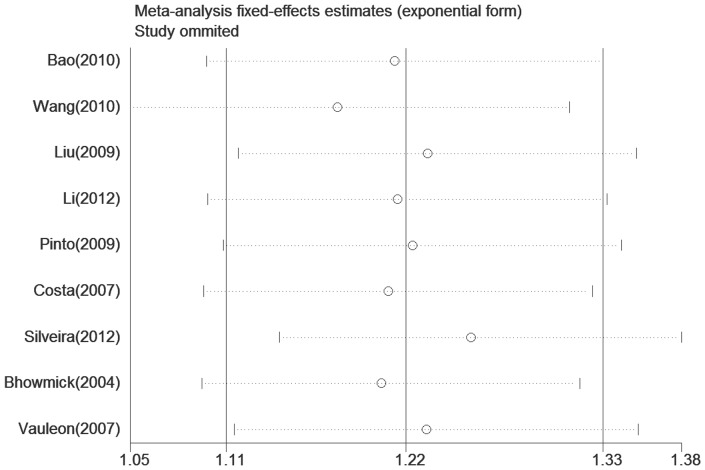
Sensitivity analysis of the association between EGF +61G/A polymorphism and glioma risk (a versus A). Results were computed by omitting each study in turn. Meta-analysis fixed-effects estimates (exponential form) were used. Open circle indicates the pooled OR, given named study is omitted. The two ends of the dotted lines represent the 95% CI.

To assess the influence of each individual study on the pooled ORs, we performed a sensitivity analysis by omitting individual study. The analysis results suggested that no individual study significantly affected the pooled ORs under the allele model (a versus A) of EGF +61G/A polymorphism (Fig. 5), which indicates that our results are statistically robust.

### Publication bias

**Figure 6:**
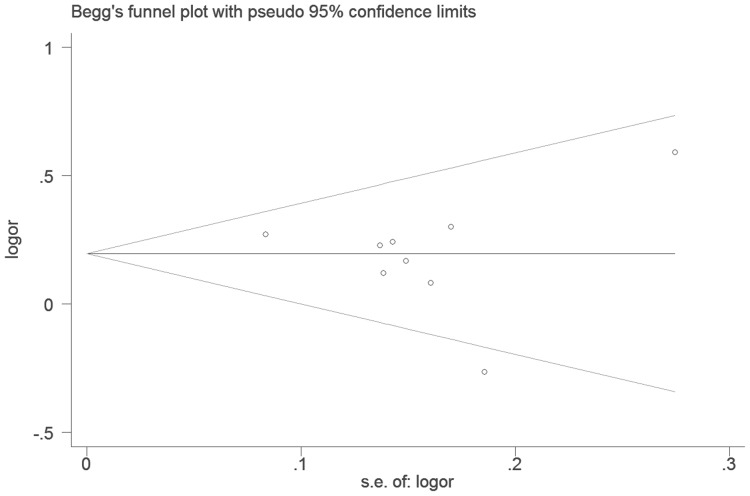
Begg's funnel plot of publication bias for EGF +61G/A polymorphism (a versus A). Plots are shown with pseudo 95% confidence limits. s.e., standard error.

Begg's funnel plot and Egger's test were performed to assess the publication bias of included literatures. The shapes of the funnel plots in all genetic models of EGF +61G/A polymorphism seemed symmetrical (Fig. 6), suggesting the lack of publication bias in the meta-analysis. Then, the Egger's test was used to provide statistical evidence of funnel plot symmetry. As expected, the results shown a significantly statistical evidence of no publication bias (t = −0.47, P = 0.656 for a versus A).

## Discussion

Cell growth dysregulation is one important characteristic of tumors. EGF/EGFR signaling dysregulation often occurs in gliomas and plays a key role in gliomagenesis [35], [36]. EGF can activate a cascade of intracellular signaling molecules, these molecules are important for cell proliferation, survival, migration, and differentiation [37]. The abnormalities of EGF/EGFR-mediated pathway are often associated with poor clinical outcome [38]. Therefore, it is logical to hypothesize that functional genetic polymorphisms involving EGF or EGFR may predispose to glioma development.

Many retrospective studies have shown that polymorphism in EGF or EGFR genes strongly correlates with susceptibility to glioma. Some studies have reported the association between polymorphism in EGF +61G/A and glioma susceptibility [20], [21], [28]–[34], but the results of these studies are not consistent. Various teams considered the inconformity is derived from different ethnicity [16], [17]. However, we hypothesize that this racial discrepancy could attribute to the diverse distribution of EGF gene frequencies among the different ethnic groups. In this meta-analysis, on the basis of collecting more studies than previous systemic review, we tried to eliminate the racial difference by defining the small letter “a” as the minor allele or the mutant allele of EGF +61G/A polymorphism, the capital letter “A” as the major allele or the common allele of EGF +61G/A polymorphism. As we expected, the analysing results showed the consistent and significant associations between the EGF +61G/A polymorphism and glioma susceptibility in in all genetic models.

From the above, we concluded that EGF +61G/A polymorphism is associated with the susceptibility to glioma. As the literature reported, EGF +61G/A polymorphism is also probably associated with the survival time of glioma patients [30]. Since glioma grade is the most informative factor for stratification into subgroups with different prognoses [5], We speculated that EGF +61G/A polymorphism may be associated with the glioma grade. Becuase many studies did not estimate the association between EGF +61G/A polymorphism and the risk of glioma at different grade. So a strict analysis of the association of EGF +61G/A polymorphism and glioma risk should be performed.

Our approach allowed us to look for potential glioma grade differences in the association between EGF +61G/A polymorphism and glioma risk. Analysis of glioma grade subgroups showed a strong association between subjects carrying at least one “a” allele with the risk of glioblastoma (Grade IV), but this association was not found in Grade I-III glioma. We further compared the EGF +61G/A polymorphism in patients with glioblastoma and Grade I-III glioma, and found that glioblastoma has a stronger association with EGF +61G/A polymorphism compared with Grade I-III glioma, this means there is a significant association between the EGF +61G/A polymorphism and the malignancy of glioma.

We think this result will strengthen the previous knowledge about EGF +61G/A polymorphism and glioma risk. This study has an important implication for glioma progress prediction that genotyping for the EGF +61G/A variant might help in identifying patients at high risk of glioblastoma. However, it needs to be emphasized that our meta-analysis involved only one study with caucasian Grade I-III glioma population, so this association should be re-evaluated in studies with larger sample sizes. Therefore, the results in the present study should be interpreted with caution.

There are some limitations that should be considered to interpret this result. Confounding factors may affect the meta-analysis. Firstly, detailed information such as age and gender in cases and controls of different genotypes in some studies were not available, which limited further analysis. Secondly, the numbers of published studies were not enough for a comprehensive analysis, particularly for different grades of glioma, we could not obtain enough statistical power to investigate the real association. Because of the limited analysing data, we can only conclude that EGF +61G/A polymorphism is related to high grade gliomas. In current results, the correlativity between EGF +61G/A polymorphism and low grade glioma is not clear. Until now, little is known about its function in primary and secondary glioblastomas. As Kunihiko Watanabe reported that immunoreactivity for the EGFR prevailed in primary glioblastomas but was rare in secondary glioblastomas [39], it infers that disturbance of EGF-EGFR signalling pathway including SNP in EGF and EGFR genes may generally participate in primary glioblastomas instead of the secondary glioblastomas. This speculation corresponds with our conclusion more or less, however, whether EGF +61G/A polymorphism mutates during the development from low grade to high grade gliomas needs further experimental explorations, more studies are needed to detect the EGF +61G/A polymorphism and their associations with those classified gliomas.

Despite of these limitations, our meta-analysis still has clear advantages over previous studies. To the best of our knowledge, this is the first meta-analysis of the association between EGF +61G/A gene polymorphisms and the susceptibility to glioma subject to different grades. In addition, data were abstracted from different studies, which significantly increased statistic power of this meta-analysis. Meanwhile, studies included in this meta-analysis were case–control studies and contained available genotype frequency, which met our selection criterions.

In summary, this meta-analysis suggested that the EGF +61G/A polymorphism is associated with glioma development risk. Additionally, we found that this association was more prominent in glioblastoma than in Grade I-III glioma, showing that the EGF +61G/A polymorphism is not only associated with the susceptibility to glioma, but also with the malignance of glioma. Future well designed larger studies are warranted to validate this association in different grade populations of glioma.

## Supporting Information

Figure S1 **Forest plot of glioma risk for EGF +61G/A polymorphism (aa/Aa versus AA).** The squares and horizontal lines correspond to the study-specific OR and 95% CI. The area of the squares reflects the study-specific weight (inverse of the variance). The diamond represents the pooled OR and 95% CI.
(TIF)

Figure S2 **Forest plot of glioma risk for EGF +61G/A polymorphism (aa/Aa versus AA) by glioma grade.** The dots and horizontal lines correspond to the study-specific OR and 95% CI. The diamond represents the pooled OR and 95% CI.
(TIF)

Figure S3 **Forest plot of EGF +61G/A polymorphism associated with glioblastoma and Grade I-III glioma risk (aa/Aa versus AA).** The squares and horizontal lines correspond to the study-specific OR and 95% CI. The area of the squares reflects the study-specific weight (inverse of the variance). The diamond represents the pooled OR and 95% CI.
(TIF)

Checklist S1 **PRISMA Checklist.**
(DOC)
