# Supplementary figures and images for: Association between the Epidermal Growth Factor +61G/A Polymorphism and Glioma Risk: A Meta-Analysis
Source: PLoS One. 2014 Apr 16;9(4):e95139. doi: 10.1371/journal.pone.0095139 (PMC3989292; doi:10.1371/journal.pone.0095139)

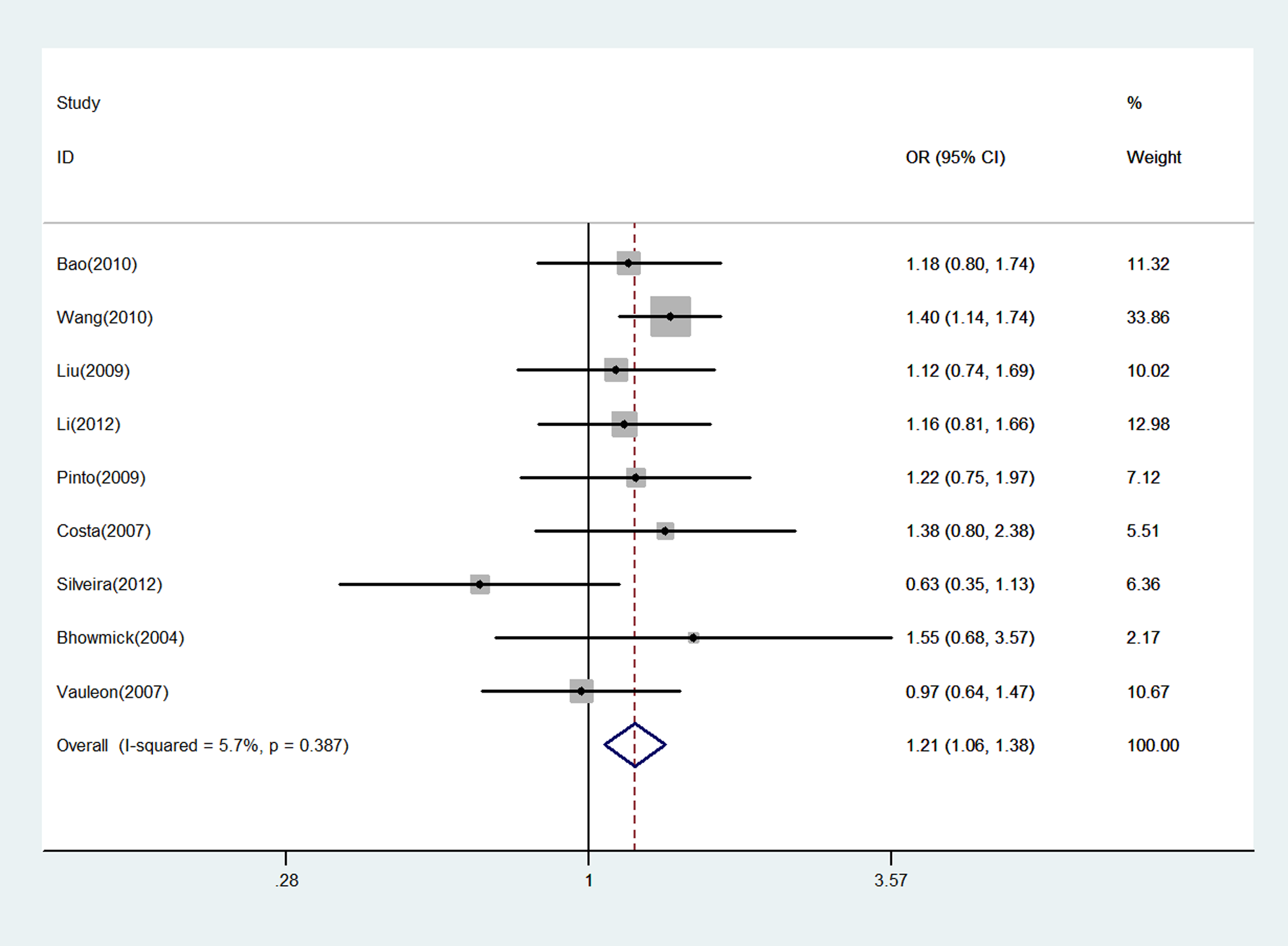

Supplement: Figure S1 — Forest plot of glioma risk for EGF +61G/A polymorphism (aa/Aa versus AA). The squares and horizontal lines correspond to the study-specific OR and 95% CI. The area of the squares reflects the study-specific weight (inverse of the variance). The diamond represents the pooled OR and 95% CI. (TIF) [file pone.0095139.s001.tif]

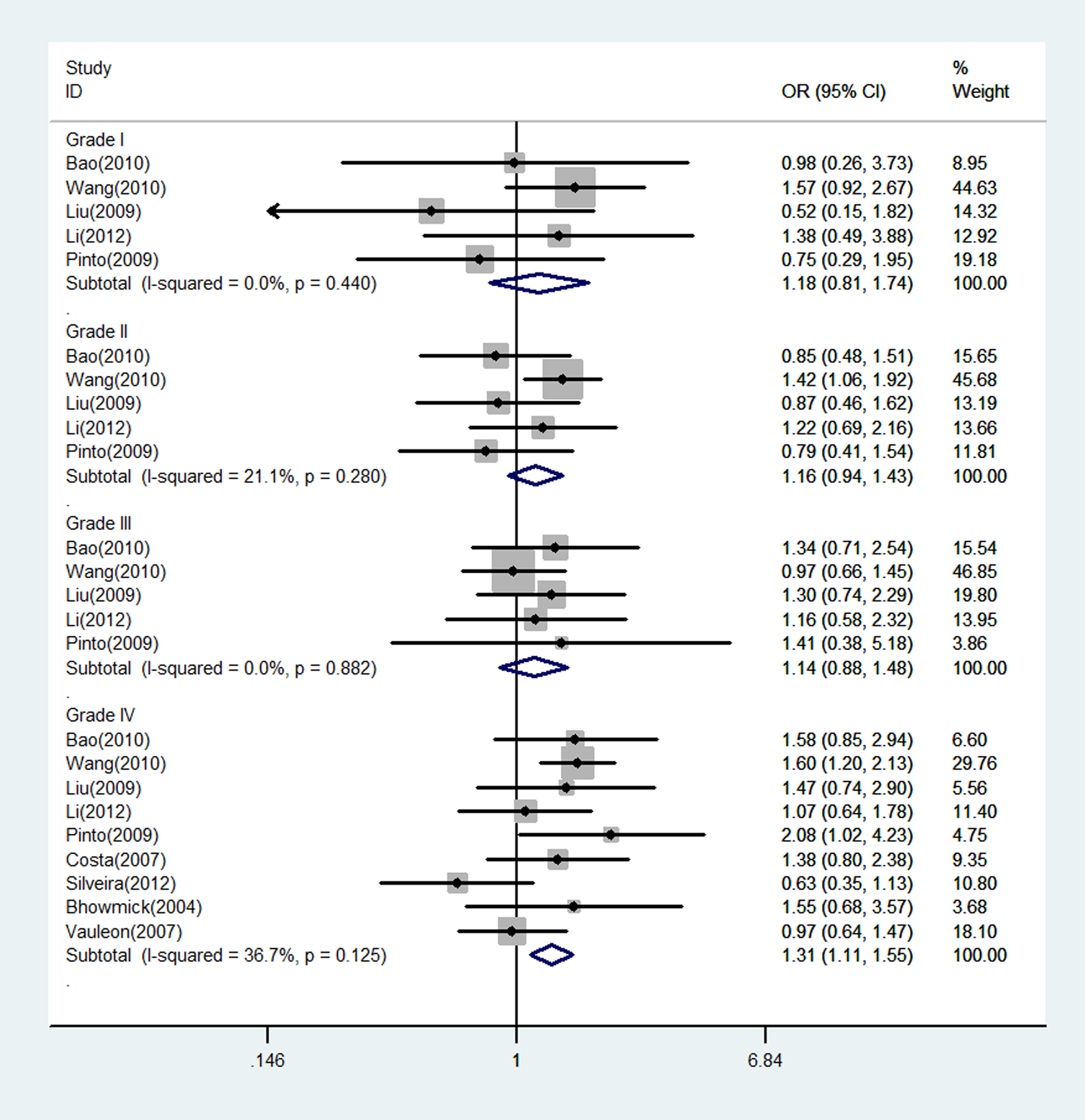

Supplement: Figure S2 — Forest plot of glioma risk for EGF +61G/A polymorphism (aa/Aa versus AA) by glioma grade. The dots and horizontal lines correspond to the study-specific OR and 95% CI. The diamond represents the pooled OR and 95% CI. (TIF) [file pone.0095139.s002.tif]

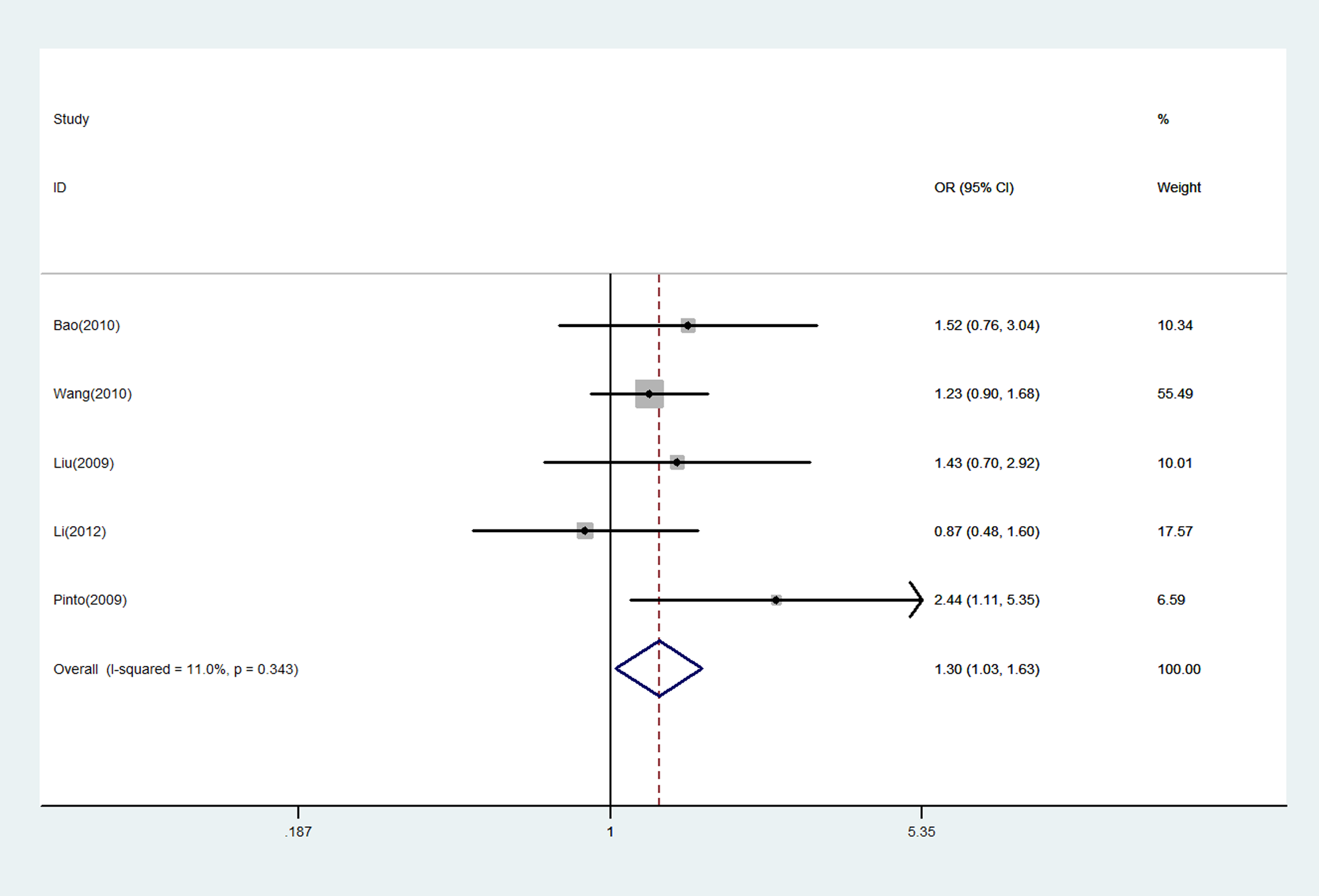

Supplement: Figure S3 — Forest plot of EGF +61G/A polymorphism associated with glioblastoma and Grade I-III glioma risk (aa/Aa versus AA). The squares and horizontal lines correspond to the study-specific OR and 95% CI. The area of the squares reflects the study-specific weight (inverse of the variance). The diamond represents the pooled OR and 95% CI. (TIF) [file pone.0095139.s003.tif]
